# Supplementary material for: CRYPTOCHROMES confer robustness, not rhythmicity, to circadian timekeeping
Source: EMBO J. 2021 Jan 25;40(7):e106745. doi: 10.15252/embj.2020106745 (PMC8013833; doi:10.15252/embj.2020106745)
Supplement: Supplementary file 1 — Appendix [file EMBJ-40-e106745-s001.docx]

**Appendix, Putker et al.**

**Table of contents:**

- **Technical Discussion**
- **Supplementary Materials and Methods**
- **Supplementary References**

**Technical Discussion**

Regarding previously reported CKO mouse locomotor rhythms

Whilst our findings are similar, the period of CKO locomotor activity we observed is shorter than that observed by Ono et al (Ono et al., 2013a, 2013b). Our methodology differed as follows: prior to behavioural recordings as adults, our CKO mice were weaned and raised under normal husbandry conditions (12h:12h light:dark cycle) whereas Ono et al. raised mice in constant light from birth; secondly, our objective analysis for period of rhythmicity employed the periodogram function of ClockLab 6.0 (significance threshold p=0.0001), whereas Ono *et al* fit regression lines by eye.

Regarding previously reported CKO PER2::LUC cellular arrhythmicity

A previous report suggests that no circadian bioluminescence rhythm in PER2::LUC activity is expressed by any tissues or cells derived from CKO mice (Liu et al., 2007). Besides our own finding, this earlier conclusion has already been contradicted for neonatal SCN slices by two independent labs (Maywood et al., 2011; Ono et al., 2013), and so it seems likely that some methodological difference must account for differences between their observations and those from other labs using the same genetic model. Critically, the CKO rhythms we observe conform to the formal definition of circadian rhythms: oscillations with (about) daily frequency, that are temperature-compensated and whose phase is sensitive to appropriate external timing cues (Pittendrigh, 1960).

Regarding the definition of circadian rhythm as applied to CKO cells, tissues and mice

A circadian rhythm is a temperature-compensated oscillation in some biological parameter of approximately daily periodicity that persists under constant conditions and is entrained by relevant environmental timing cues. The bioluminescence oscillations observed in CKO cells and tissues conform to this long-established definition (Pittendrigh, 1960). For mammalian systems, the fundamental capacity to maintain a circadian rhythm (circadian timekeeping) is a cellular phenomenon, in that isolated cells are the most reduced system where circadian rhythms are observed. It follows that any higher level circadian function arises from the combination of cellular timekeeping and communication between cells. Co-ordinated circadian rhythms in behaviour and physiology are observed in multi-cellular mammals under non-stressed conditions when individual cellular rhythms are synchronised; and frequently amplified by appropriate extrinsic and intrinsic timing cues. Thus whether or not the short (~17h) locomotor rhythms observed in CKO mice, after transition from constant light to constant dark, can be described as “circadian” boils down to whether or not they can be considered to entrain to environmental timing cues and whether or not 17h can be described as “approximately daily”. The latter is a rather philosophical question and cannot be satisfactorily resolved here. Whereas, the inability of CKO mice to entrain to 12h:12h light:dark cycles argues against the former, but does not preclude the possibility that rhythms in CKO mice might entrain to different zeitgebers, or zeitgebers of different periodicity, or normally be masked rather than absent.

Addressing the first, as noted in the discussion, CKO mice do entrain to daily feed-fast cycles, and behavioural rhythms can be synchronised by the transition from constant light to constant darkness. Considering the second, it seemed plausible that CKO mice might either entrain to a short 8h:8h light:dark (16h day) due to an intrinsically shorter endogenous clock or else have a general deficiency to entrainment by light:dark cycles. We tested this, and data in Figure EV2 support the latter possibility, in that neither WT nor CKO mice stably entrained to 16h cycles whereas WT, but not CKO, mice entrained to 24h days.

We note, however, that the short period locomotor rhythms observed in CKO mice under quite specific environmental conditions correlate strongly with the *bona fide* short period circadian rhythms observed in CKO cells and SCN. Moreover, in a separate study we found that CKO cells show increased basal stress and sensitivity to stress that has profound effects on protein homeostasis (Wong et al, 2020). Overall then, we consider these observations to be consistent with persistence of the capacity to maintain behavioural rhythms close to the circadian range in CKO mice, that is masked under most circumstances and is not engaged by environmental light:dark cycles. We suggest that *in vivo* the (pathophysiological) stress of CRY-deficiency is epistatic to the expression of daily locomotor activity rhythms following entrainment by standard light:dark cycles; and that this arises through impaired communication between cells rather than because those cells intrinsically lack circadian timekeeping. Thus, whilst not arrhythmic, CKO mouse locomotor rhythms also cannot be described as circadian in the strictest sense.

**Supplementary Materials and Methods**

**Entrainment protocols for cell experiments**

Fibroblast bioluminescence recordings were performed with confluent, quiescent monolayers. Due to iterative refinement of the synchronisation protocol used to optimise rhythms in the CKO fibroblasts, several different entrainment protocols were used throughout the paper, with appropriate WT controls. We found the most effective method employed temperature entrainment cycles (12h 32°C – 12h 37°C) for at least five days, prior to incubation of cells with 100 nM dexamethasone for 2 h beginning at 4 h after the start of the warm phase, followed immediately by changing cells into the experimental recording medium. Despite our best efforts, over many years, we were unable to identify a set of entrainment and recording conditions that consistently produced CKO PER2::LUC rhythms and were forced to conclude more variables were at play than we were adequately able to control for. Those that we tested are as follows: seeding density, passage number, glucose concentration, serum concentration, amino acid concentration, time in culture, time in temperature cycles, period length of temperature cycles, pH buffer, growth factor (B27) concentration, co-culture with WT cells and media conditioning. A more detailed description can be found online: https://www.repository.cam.ac.uk/handle/1810/300610. To reduce variation in cell attachment as a source of experimental error we used a fibronectin coating in some experiments; this did not significantly affect subsequent bioluminescence rhythms.

The following entrainment protocols were used for the following experiments:

- 1E, F, S1C, E-G: combination of all the entrainment methods described above
- 2A: medium change at the indicated timepoints (arrow)
- 2C: 12h:12h 32^o^C:37^o^C temperature entrainment only
- 3A: Prior 12h:12h 32^o^C:37^o^C temperature cycles
- 3B: Prior 12h:12h 32^o^C:37^o^C temperature cycles
- 3C/S3C: Prior temperature cycles and media change
- 3D/S3E-H: Prior temperature cycles and media change
- 4A: Prior temperature cycles, dexamethasone pulse and media change
- S4A: Prior temperature cycles, dexamethasone pulse and media change
- 4D-H: Prior temperature cycles, dexamethasone pulse and media change
- 5A-B: Prior temperature cycles and media change
- 5C: Prior temperature cycles, dexamethasone pulse and media change
- S5D: Prior temperature cycles and media change

**PER2 molecule count**

Cells were seeded in 10% DMEM and entrained by temperature cycles for three days after which they were shifted to constant 37°C**.** At the following estimated peak of PER2::LUC activity (as reported in a co-recording), cells were trypsinised and washed in PBS, after which 5 x10^6^ cells were lysed in 100 mM potassium phosphate buffer pH 7.8, 1 mM EDTA, 100 mM 2-mercaptoethanol, 1% triton, 10% glycerol and protease inhibitors. Lysates were cleared by centrifugation and diluted tenfold in 15 mM MgSO_4_, 30 mM HEPES, 300 µM luciferin and 1 mM ATP. Dilutions were staggered to correct for time lags between bioluminescence measurements. PER2::LUC activity was measured in a plate reader and the number of molecules was calculated by comparing the bioluminescence signal to a standard curve of QuantiLum® Recombinant Luciferase (Promega) which was spiked into (luciferase-free) cell lysate to control for factors in the lysate that might affect bioluminescence. Three technical replicates were measured in every experiment and the experiment was carried out three times. A representative experiment is shown.

**qPCR time course**

Cells were seeded in 10% DMEM and entrained by temperature cycles over five days. 24 hours before the first time point, media was changed to 1% air medium (1% serum, +1 mM luciferin), after which the cells were placed in constant conditions (37°C) and the recording begun. Cells were harvested in triplicate every four hours from 24 hours up to 48 hours after media change. Cells were harvested in RLT buffer according to protocol from the Qiagen RNeasy kit and samples were immediately snap frozen. mRNA isolation was performed according to the manufacturer’s protocol (including DNase treatment), as was cDNA synthesis (Biorad iScript™ cDNA Synthesis Kit). qPCR was performed using SYBR® FAST qPCR Kit (KAPA biosystems) on a Prime Pro 48 Real-time qPCR machine (Bibby Scientific) (for primer and protocol details see below). Analysis involved three technical and three biological replicates. Relative amounts of mRNA were determined by comparing the samples to a standard curve, and expressed relative to ribosomal RNA Rns18s.

**Primers**

Primers used for genotyping CRY1/2:

| Gene name | Genotype | Primer | Temp |
| --- | --- | --- | --- |
| *Cry1* | WT | CAGGAGGAGAAACTGAGGCACT | 63°C |
|  | CKO | TGAATGAACTGCAGGACGAG |  |
|  | WT/CKO | GTGTCTGGCTAAATGGTGG |  |
| *Cry2* | WT | CCAGAGACGGGAAATGTTCTT | 57°C |
|  | CKO | GAGATCAGCAGCCTCTGTTCC |  |
|  | WT/CKO | GCTTCATCCACATCGGTAACTC |  |

Primers used for QPCR:

| Gene name | Forward primer | Reverse primer | Temp |
| --- | --- | --- | --- |
| *Rns18s* | CGCCGCTAGAGGTGAAATTC | TTGGCAAATGCTTTCGCTC | 58°C |
| *Bmal1* | ACGACATAGGACACCTCGCAGA | CGGGTTCATGAAACTGAACCATC | 55°C |
| *Per2* | CCTACAGCATGGAGCAGGTTGA | TTCCCAGAAACCAGGGACACA | 58°C |

**Puromycin labelling time course**

Wild type and CRY-deficient PER2::LUC cells were seeded in fibronectin-coated 6-well plates in 10% DMEM and temperature entrained for five days. 18 hours before harvest they were pulsed with dexamethasone (100 nM) for 2 hours, and changed into air medium (HEPES, with 1 mM luciferin), after which the bioluminescence recording commenced. Cells were harvested every 4 hours (three biological replicates per time point) directly from the recording device from 16 hours up to 64 hours after media change. Ten minutes before harvest, cells were placed on a 37°C heat-pad and pulsed with 10 µg/mL puromycin. Cells were washed in ice-cold PBS, 5 mM EDTA and 20 mM NEM, harvested in 50 mM Tris-HCl pH 7.4, 150 mM NaCl, 0.1% LDS, 1% Triton, 0.5 % NaDOC, 20mM NEM and protease inhibitors, with samples then being immediately frozen in liquid nitrogen. After thawing, samples were cleared by centrifugation and taken up in reducing LDS sample buffer for Western blot analysis. Puromycin incorporation was assayed by Western blotting with specific anti-puromycin antibody (PMY-2A4-2, Developmental studies hybridoma bank). The signal was corrected for total protein loading on coomassie blue staining.

**Acute PER2::LUC measurements**

WT and CKO cells PER2::LUC cells were seeded in absence of luciferin in fibronectin-coated 35 mm dishes and synchronised by temperature entrainment, dexamethasone pulse and change into air medium. Cells for parallel co-recordings were pre-incubated with 0.1 mM luciferin. Cells were harvested every hour (in triplicate) from the recording incubator from 16 hours up to 64 hours after media change. Cells were washed in ice-cold PBS, lysed in 100 mM potassium phosphate buffer pH 7.8, 1 mM EDTA, 7 mM 2-mercaptoethanol, 1% triton, 10% glycerol, 1 mM NaF, 1 mM Na_3_VO_4_ and protease inhibitors, then samples were immediately frozen in liquid nitrogen. After thawing, samples were cleared by centrifugation and used for acute luciferase assays. Bioluminescence activity in 10 µL sample was measured in triplicate in a Spark 10M microplate reader (Tecan) and initiated by injection of 90 µL1.5 mM MgSO_4_, 30 mM HEPES, 300 µM luciferin and 1 mM ATP. Immediate reading obviated the need for differential times of incubation prior to measurement.

**Co-immunoprecipitation experiments**

For assaying the interaction between BMAL1 and PER2::LUC, cells were entrained in temperature cycles for 4 days and harvested directly from temperature cycles at the expected peak of PER2::LUC expression (4 hours after change to 32°C). Cells were washed in ice-cold PBS and lysed in 200 µL 50 mM Tris-HCl pH 7.5, 1% TX100, 10 mM MgCl_2_, 100 mM NaCl, DNase (100 U/mL) and protease inhibitors. After incubation at 4°C for 10 minutes, lysates were passed through a 19 gauge needle to ensure complete lysis, cleared by centrifugation, and diluted in 1 mL ice-cold wash buffer (50 mM Tris-HCl pH 7.5, 0.1% TX100, 5mM EDTA,1.5 mM MgCl_2_, 100 mM NaCl and protease inhibitors). Total lysate samples were taken and stored at 4°C for the duration of the experiment. BMAL1 was precipitated with goat-anti-BMAL1 (Santa Cruz, SC-8550) antibodies or control IgG (SC-2028) coupled to protein G agarose beads (Pierce 20398) while rocking for two hours at 4°C. Samples were washed 3 times with ice-cold wash buffer and once in minimal luciferase assay buffer (15 mM MgSO4, 30 mM HEPES). PER2::LUC co-immunoprecipitation (co-IP) was measured in a luciferase assay by mixing the beads in 200 uL luciferase assay buffer (15 mM MgSO4, 30 mM HEPES, 300 µM luciferin, 1 mM ATP, 10 mM 2-mercaptoethanol) and measuring luciferase activity in a Berthold platereader. The results were corrected for input (5 µL of the total lysate sample) and plotted relatively to the WT IgG pulldown. After measurements, the beads were washed in wash buffer and taken up in sample buffer for Western blot analysis of BMAL1 pull-down efficiency (homemade rabbit-anti-BMAL1 antibody (Sládek et al., 2007)). To study the interaction of BMAL1 with S6K and eIF4, cells were entrained by a 2-hour dexamethasone pulse, after which they were changed into normal growth medium. 12 and 24 hours after the medium change, BMAL1 immunoprecipitation was executed as described above. After washing with wash buffer, samples were taken up in reducing LDS sample buffer and analysed by Western blot for presence of BMAL1, S6K and eIF4 (Cell Signaling, resp. #2708 and #2013)

**Half-life determination experiments**

Half-life of PER2::LUC and SV40::LUC were determined by following luciferase decay upon treatment with saturating concentrations of CHX (10 μM) which was pulsed in at pre-set times-of-day. Cells were synchronised by temperature entrainment, dexamethasone pulse and change into air medium.

For comparing effects of SV40::LUC levels on protein decay, multiple stable SV40::LUC fibroblast lines were generated by puromycin selection, grown to confluence in 96-well plates, then treated with 25 μg/mL CHX at the beginning of the recording after which luciferase decay was followed over time. Random genomic integration of SV40:Luc leads to a broad range of different levels of luciferase expression, evident from the broad range of initial luciferase activities.
Half-life was determined in Prism by fitting a simple one-phase exponential decay curve.

***Drosophila* experiments**

**Fly stocks and husbandry**

The XLG-luc construct contains a fusion between Period coding sequence and Luciferase under endogenous control of the *period* promoter and flanking regulatory regions, as described previously. The luciferase signal from the derivative fly strain, *y w;; XLG-luc:2/TM3,* faithfully reports the endogenous PERIOD protein rhythm (Veleri et al., 2003).

**Generation of *tim^Out^* fly**

*Timeless* knock-out fly lines (*tim^KO^*) were generated by homologous recombination (Huang et al., 2009) and described in (Lamaze et al., 2017). The residual mini-*white* marker in the *tim^KO^* flies was floxed-out. The obtained knockout lines were denoted as *w^1118^; tim^Out^*. One derivative strain, *y w; tim^Out^/CyO; XLG-luc:2/TM3*, was generated via standard balancer crossing.

**Longitudinal XLG-luciferase recordings**

To monitor circadian rhythm of PER::LUC activity, we crossed male *y w;; XLG-luc:2/TM3 and y w; tim^Out^/CyO; XLG-luc:2/TM3* flies with two female control*, w^1118^* and *Canton S*, and to two clock mutants, *per^01^* and  *w^1118^; tim^Out^*, respectively. The resultant F1 males: *per^01^/Y*;+/+;*XLG-luc:2/+*,*w^1118^/Y*;+/+;*XLG-luc:2/+*,*+^Canton S^/Y ;+/+;XLG-luc:2/+*,  and*w^1118^/Y;tim^Out^/tim^Out^;XLG-luc:2/+*were studied.

Three to seven day-old flies were then entrained for three day LD cycles before being loaded individually into the wells of a microtiter plate containing the food-luciferin substrate (15mM luciferin) where their movement was restricted by covering them with pierced plastic domes (Stanewsky et al., 1997). Recordings were performed under constant darkness at 26°C over seven days. Bioluminescence images were recorded with contiguous 5 min integrations over 7 days with camera settings. The plate was placed in the ALLIGATOR and recorded in parallel with the tube-based assay condition. Bioluminescence from each fly was background subtracted, summed into 2-hour bins, and then detrended using a 24-hour moving average. Since the per^01^ and control XLG-luc flies produced very similar bioluminescence traces, for clarity only the *tim^Out^* and WT control data are shown*.*

**Mathematical modeling**

For the mathematical model, we assumed that PER2::LUC translation at time (t) is a function of *Per2*::*Luc* mRNA abundance, corrected for the changes we observed for global translation rate over time; and that PER2::LUC degradation rate follows one-phase exponential decay kinetics where the decay constant is defined by a sine wave with 24-hour periodicity, with the amplitude, phase and other parameters being derived entirely from experimental measurements, as follows:

P_t_ = P_t-1_ + S_t_ - D_t_

S_t_ = 1000*R_t_*T

D_t_ = P * (1- e^-kt^)

k_t_ = ln(2)/((A(sin((2π*t/24)+φ)))+H)

t = time in hours

P_t_ is PER2::LUC protein abundance at t (from measured number of molecules/cell)

S_t_ is total P translated in 1 h prior to t

D_t_ is total P degradation in 1 h prior to t

R_t_ is *Per2*::*Luc* mRNA abundance at t (interpolated from qRT-PCR measurements)

T_t_ is translation rate at t (interpolated from puromycin incorporation assays)

k_t_ is the exponential decay constant at t (from PER2::LUC half-life measurements)

A is amplitude of the rhythm in PER2::LUC half-life (from PER2::LUC half-life measurements)

φ = initial phase (from observed phase of PER2::LUC relative to *Per2*::*Luc* mRNA level)

H = is mean PER2::LUC half-life (from PER2::LUC half-life measurements)

**Supplementary references**

Beale, A.D., Kruchek, E., Kitcatt, S.J., Henslee, E.A., Parry, J.S.W., Braun, G., Jabr, R., von Schantz, M., O’Neill, J.S., and Labeed, F.H. (2019). Casein Kinase 1 Underlies Temperature Compensation of Circadian Rhythms in Human Red Blood Cells. J. Biol. Rhythms *34*, 144–153.

Chen, R., Seo, D.O., Bell, E., Von Gall, C., and Lee, D.C. (2008). Strong resetting of the mammalian clock by constant light followed by constant darkness. J. Neurosci. *28*, 11839–11847.

Gallego, M., and Virshup, D.M. (2007). Post-translational modifications regulate the ticking of the circadian clock. Nat. Rev. Mol. Cell Biol. *8*, 139–148.

Hara, K., Tydeman, P., and Kirschner, M. (1980). A cytoplasmic clock with the same period as the division cycle in Xenopus eggs. Proc. Natl. Acad. Sci. U. S. A.

Huang, J., Zhou, W., Dong, W., Watson, A.M., and Hong, Y. (2009). From the Cover: Directed, efficient, and versatile modifications of the Drosophila genome by genomic engineering. Proc. Natl. Acad. Sci. U. S. A. *106*, 8284–8289.

Iijima, M., Yamaguchi, S., van der Horst, G.T.J., Bonnefont, X., Okamura, H., and Shibata, S. (2005). Altered food-anticipatory activity rhythm in Cryptochrome-deficient mice. Neurosci. Res. *52*, 166–173.

Lakin-Thomas, P.L. (2006). Transcriptional feedback oscillators: maybe, maybe not... J. Biol. Rhythms *21*, 83–92.

Lamaze, A., Öztürk-Çolak, A., Fischer, R., Peschel, N., Koh, K., and Jepson, J.E.C. (2017). Regulation of sleep plasticity by a thermo-sensitive circuit in Drosophila. Sci. Rep.

Liu, A.C., Welsh, D.K., Ko, C.H., Tran, H.G., Zhang, E.E., Priest, A. a., Buhr, E.D., Singer, O., Meeker, K., Verma, I.M., et al. (2007). Intercellular Coupling Confers Robustness against Mutations in the SCN Circadian Clock Network. Cell *129*, 605–616.

Meng, Q.-J.J., Logunova, L., Maywood, E.S., Gallego, M., Lebiecki, J., Brown, T.M., Sládek, M., Semikhodskii, A.S., Glossop, N.R.J., Piggins, H.D., et al. (2008). Setting clock speed in mammals: the CK1 epsilon tau mutation in mice accelerates circadian pacemakers by selectively destabilizing PERIOD proteins. Neuron *58*, 78–88.

O’Neill, J.S., and Reddy, A.B. (2011). Circadian clocks in human red blood cells. Nature *469*, 498–503.

O’Neill, J.S., and Reddy, A.B. (2012). The essential role of cAMP / Ca 2 + signalling in mammalian circadian timekeeping. Biochem Soc Trans *40*, 44–50.

O’Neill, J.S., Ooijen, G. Van, Dixon, L.E., Troein, C., Corellou, F., Bouget, F.-Y., Reddy, A.B., and Millar, A.J. (2011). Circadian rhythms persist without transcription in a eukaryote. Nature *469*, 554–558.

Ono, D., Honma, S., and Honma, K. (2013a). Cryptochromes are critical for the development of coherent circadian rhythms in the mouse suprachiasmatic nucleus. Nat. Commun. *4*, 1666.

Ono, D., Honma, S., and Honma, K.-I. (2013b). Postnatal constant light compensates Cryptochrome1 and 2 double deficiency for disruption of circadian behavioral rhythms in mice under constant dark. PLoS One *8*, e80615.

van Ooijen, G., Dixon, L.E., Troein, C., and Millar, A.J. (2011). Proteasome function is required for biological timing throughout the twenty-four hour cycle. Curr. Biol. *21*, 869–875.

Pittendrigh, C.S. (1960). Circadian rhythms and the circadian organization of living systems. Cold Spring Harb. Symp. Quant. Biol. *25*, 159–184.

Pomerening, J.R., Sun, Y.K., and Ferrell, J.E. (2005). Systems-level dissection of the cell-cycle oscillator: Bypassing positive feedback produces damped oscillations. Cell *122*, 565–578.

Ptáček, L.J., Jones, C.R., and Fu, Y.H. (2007). Novel insights from genetic and molecular characterization of the human clock. Cold Spring Harb. Symp. Quant. Biol. *72*, 273–277.

Putker, M., and O’Neill, J.S. (2016). Reciprocal Control of the Circadian Clock and Cellular Redox State - a Critical Appraisal. Mol. Cells *39*, 6–19.

Sládek, M., Rybová, M., Jindráková, Z., Zemanová, Z., Polidarová, L., Mrnka, L., O’Neill, J., Pácha, J., and Sumová, A. (2007). Insight Into the Circadian Clock Within Rat Colonic Epithelial Cells. Gastroenterology *133*, 1240–1249.

Stanewsky, R., Jamison, C.F., Plautz, J.D., Kay, S.A., and Hall, J.C. (1997). Multiple circadian-regulated elements contribute to cycling period gene expression in Drosophila. EMBO J. *16*, 5006–5018.

Sweeney, B., and Haxo, F. (1961). Persistence of a Photosynthetic Rhythm in Enucleated Acetabularia. Science (80-. ). *134*, 1361–1363.

Top, D., O’neil, J.L., Merz, G.E., Dusad, K., Crane, B.R., and Young, M.W. (2018). CK1/doubletime activity delays transcription activation in the circadian clock. Elife *7*, 1–21.

Veleri, S., Brandes, C., Helfrich-Förster, C., Hall, J.C., and Stanewsky, R. (2003). A Self-Sustaining, Light-Entrainable Circadian Oscillator in the Drosophila Brain. Curr. Biol. *13*, 1758–1767.

Welsh, D.K., Takahashi, J.S., and Kay, S.A. (2010). Suprachiasmatic nucleus: cell autonomy and network properties. Annu. Rev. Physiol. *72*, 551–577.

Wong, D.C., and O’Neill, J.S. (2018). Non-transcriptional processes in circadian rhythm generation. Curr. Opin. Physiol. *5*, 117–132.

Yamaguchi, S., Isejima, H., Matsuo, T., Okura, R., Yagita, K., Kobayashi, M., and Okamura, H. (2003). Synchronization of Cellular Clocks in the Suprachiasmatic Nucleus. Science (80-. ). *302*, 1408–1412.
