# Supplementary material for: CRYPTOCHROMES confer robustness, not rhythmicity, to circadian timekeeping
Source: EMBO J. 2021 Jan 25;40(7):e106745. doi: 10.15252/embj.2020106745 (PMC8013833; doi:10.15252/embj.2020106745)
Supplement: Supplementary file 2 — Expanded View Figures PDF [file EMBJ-40-e106745-s004.pdf]

Expanded View Figures

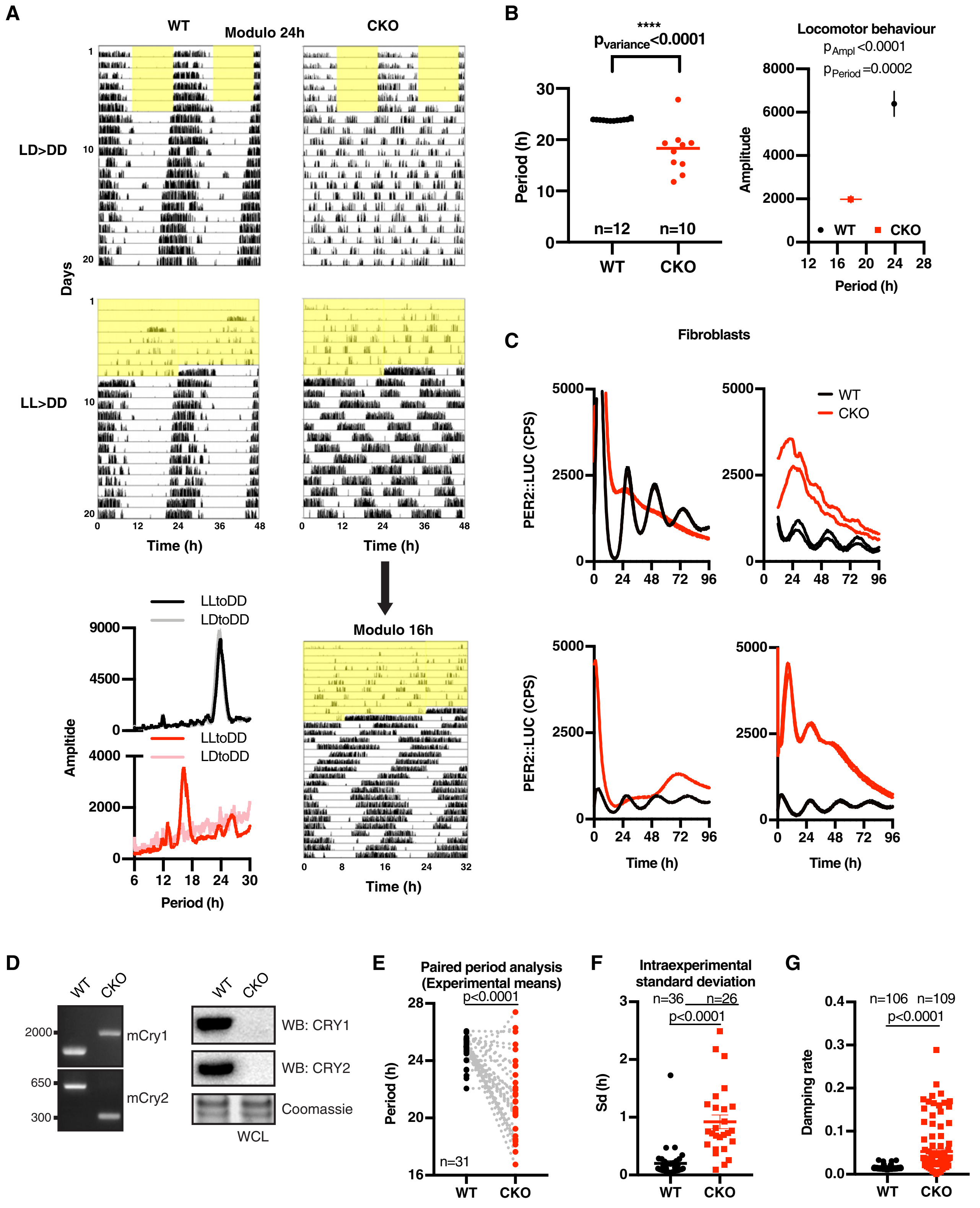

Figure EV1.

 © 2021 The Authors

Figure EV1. CRY-independent circadian timekeeping occurs cell-autonomously.

A Representative double-plotted actograms showing wheel-running activity of WT and CKO mice during 12 h:12 h light:dark (LD) cycles (yellow shading indicating lights on) (top) or during constant light (LL) (bottom) and thereafter in constant darkness (DD). Top four figures have same *x*-axis (modulo 24 h). Rhythmic behaviour of CKO mice in LL > DD condition becomes clear when plotting the data in 16-h modulo (i.e. *x*-axis being 32 h). LD figure WT modulo 24-h and CKO modulo 16-h are also presented in Fig 1A. Bottom left, representative periodograms of WT and CKO mice over 2 weeks in constant darkness, following either 12 h:12 h light:dark cycles or constant light.

B A second experimental cohort highlights significant differences in period variance (left, horizontal line represents mean, *F*-test for variance); period and amplitude (right) of CKO ($n = 10$) vs. WT ($n = 12$) mice over 2 weeks in constant darkness following 1 week under constant light (mean ± SEM, 2-way ANOVA with Sidak's MCT).

C Examples of independent bioluminescence recordings of PER2::LUC expression in CRY-deficient fibroblasts showing variability in shape and baseline of rhythmic CKO traces. Two representative traces are shown per experiment. Stringent entrainment, e.g. with temperature cycles or dexamethasone, increases the likelihood of observing rhythmicity, but only in approximately 30% of the experiments did we observe clearly rhythmic expression of PER2::LUC over 3 cycles. Despite our best efforts, over many years, we were unable to identify a set of entrainment and recording conditions that consistently produced CKO PER2::LUC rhythms and we were forced to conclude that more variables were at play than we were adequately able to control for.

D Genotyping CKO fibroblasts used throughout this study. Left: PCR genotyping shows the expected pattern of CRY1 and CRY2 knockout. Right: Western blot analysis of whole cell lysates (WCL) and probed with antibodies against CRY1 and CRY2.

E Interexperimental comparison of PER2::LUC periods in WT vs. CKO fibroblasts. Paired comparison of period means of experiments used for Fig 1F where CKO traces were rhythmic. *P*-values were calculated by paired *t*-test.

F Intraexperimental standard deviations (i.e. between replicates) were calculated for all experiments with > 3 rhythmic traces (mean ± SEM). *P*-value was calculated by unpaired *t*-test.

G Damping rates of all individual detrended traces of example experiments shown in Fig EV1E were calculated by damped sine wave fitting (mean ± SEM, WT $n = 106$, CKO $n = 109$). *P*-value was calculated by unpaired *t*-test with Welch correction.

Source data are available online for this figure.

Figure EV2. Entrainment deficiency of CRY-deficient mice to environmental light:dark cycles and Timeless-independent protein rhythms in *Drosophila melanogaster*.

A Representative actograms showing that CKO mice ($n = 5$) cannot be entrained to 8 h:8 h or 12 h:12 h light:dark cycles, whereas WT mice entrain to 12 h:12 h but not 8 h:8 h light-dark cycles ($n = 5$). Equal numbers of age-matched male and female mice were used.

B, C For CKO mice during under LD cycles, the dominant period of behavioural rhythms is determined by the period of the zeitgeber, whereas amplitude does not vary significantly between 16-h and 24-h cycles. In contrast, the period of WT behavioural rhythms does not vary significantly between 16-h and 24-h cycles, whereas amplitude is significantly reduced under unnatural 8 h:8 h light:dark cycles. This indicates the robustness conferred by CRY to circadian rhythms of locomotor activity in WT mice *in vivo*. Two-way ANOVA *P*-values and significance from Sidak's multiple comparisons test are reported; horizontal line represents mean.

D Normalised, detrended bioluminescence recording of the XLG-luciferase reporter (XLG-LUC; equivalent of PER2::LUC) expressed in WT and *timeless* knockout (Tim^{out}) flies under constant darkness (detrended means ± SEM; WT $n = 21$, Tim^{out} $n = 36$). Note the difference in *y*-axis scaling.

E Damped sine wave fit to the data presented in (D). *P*-values (extra sum-of-squares *F*-test) indicate comparison of fit test with the null hypothesis (straight line).

F Significant differences in the period and amplitude of XLG-LUC rhythms of Tim^{out} compared with WT flies. Mean ± SEM, *P*-values indicate unpaired *t*-test, WT $n = 21$, Tim^{out} $n = 36$.

Data information: The generation of Tim^{out} flies is reported in Lamaze *et al* (2017). Similar to CRY-deficient mice, whole gene *timeless* knockout flies are characterised as being behaviourally arrhythmic under constant darkness following entrainment by light:dark cycles: https://opus.bibliothek.uni-wuerzburg.de/frontdoor/index/index/year/2015/docId/11914

© 2021 The Authors

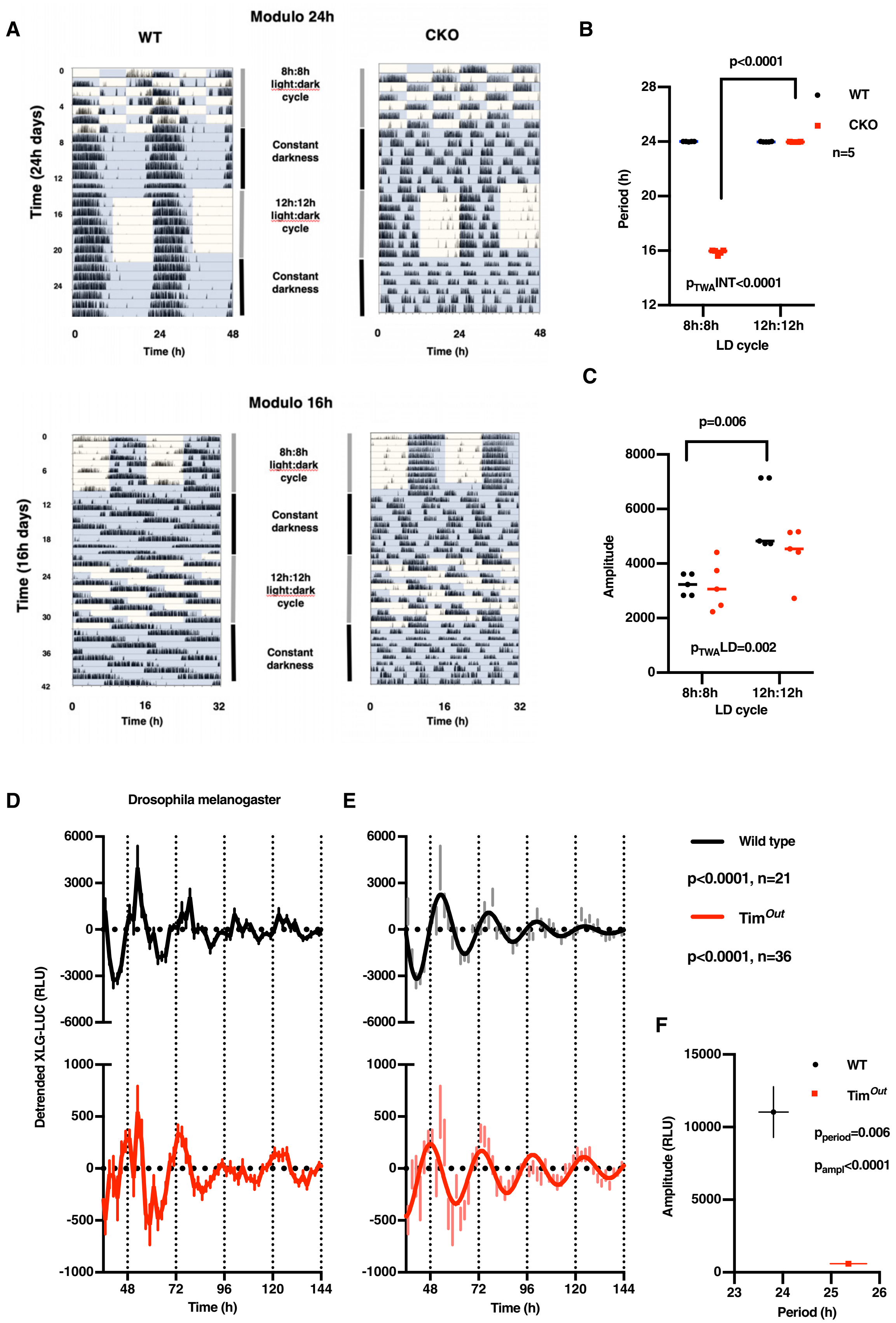

Figure EV2.

 © 2021 The Authors

Figure EV3. CRY-independent rhythms are regulated post-transcriptionally.

A Standard curve of recombinant luciferase that was used to determine the number of PER2::LUC molecules. Known concentrations of recombinant luciferase were spiked into (non-luciferase containing) cell lysate to reproduce experimental conditions, and the luciferase signal was measured with a 20-s integration time (CP 20 s; counts per 20 s). Data were fitted with a straight line (red line, ± 95% CI). The grey dotted lines indicate the (linear) area of the curve used to determine the number of PER2::LUC molecules of the experiment shown in Fig 3A.

B Western blot analysis of BMAL1 co-immunoprecipitation samples shown in Fig 3B. BMAL1 or control (IgG) pulldowns were performed at the peak of PER2::LUC expression (determined in parallel PER2::LUC recordings) in 3 technical replicates (A–C). Full blots are shown in Source data Fig EV3.

C *Bmal1* mRNA levels were determined by qPCR over one circadian period ($n = 2$, mean ± SEM). The WT timeseries were preferentially fit with a circadian damped sine wave compared with a straight line (extra sum-of-squares *F*-test, $P = 0.0321$), but not the CKO timeseries (ns). PER2::LUC co-recording from parallel cultures is depicted in Fig 3C.

D Detrended bioluminescence data of transcriptional reporter *Cry1*:LUC in WT and CKO mouse adult fibroblasts (MAFs) ($n = 4$, mean ± SEM). WT traces fit circadian damped sine wave over straight line in extra sum-of-squares *F*-test ($P < 0.0001$), whereas no sine wave could be fit to CKO traces (no *P*-value).

E Detrended *Per2* and *Nr1d1* promoter activity in WT, CKO and quadruple *Cry1/2-Per1/2* knockout (CPKO) mouse embryonic fibroblasts (MEFs) recorded at 32°C, $n = 3$, mean (solid) ±SEM (dashed). WT *Per2* and all *Nr1d1* traces were preferentially fit with a circadian damped sine wave over straight line (extra sum-of-squares *F*-test, $P < 0.0001$), whereas no sine wave could be fit to the other traces (not significant, no *P*-value). Period analysis shows that *Nr1d1* promoter oscillations are temperature-compensated (mean ± SEM, $n = 3$).

F Expanded view of *Per2*:LUC recordings to show no circadian oscillations of *Per2* promoter activity is detected in C(P)KO MEFs, $n = 3$, mean (solid) ±SEM (dashed). Zoomed-out version of 37°C recording is also shown in Fig 3D and of 32°C recording in Fig EV3E. WT *Per2* traces were preferentially fit with a circadian damped sine wave over straight line (extra sum-of-squares *F*-test, $P < 0.0001$), whereas no sine wave could be fit to the other traces (not significant, no *P*-value).

G Period analysis shows that also WT *Per2* promoter oscillations are temperature-compensated, as expected ($n = 3$, mean ± SEM).

H 24-h average (from 24 to 48 h) raw luciferase counts from *Per2*:LUC and *Nr1d1*:LUC counts demonstrate that reporter expression levels do not explain a difference in amplitude in circadian oscillations ($n = 3$, mean ± SEM).

Source data are available online for this figure.

© 2021 The Authors

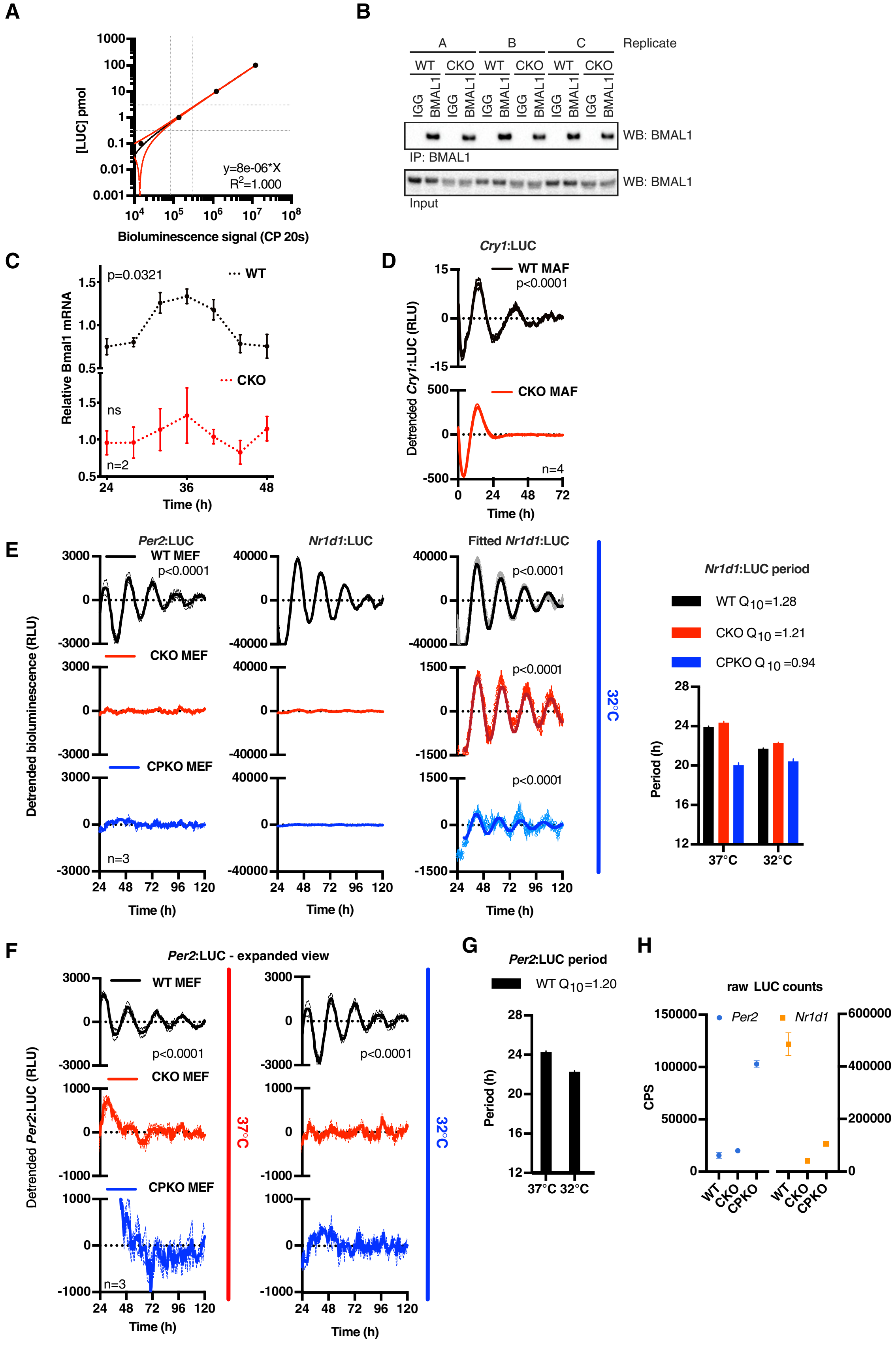

Figure EV3.

 © 2021 The Authors

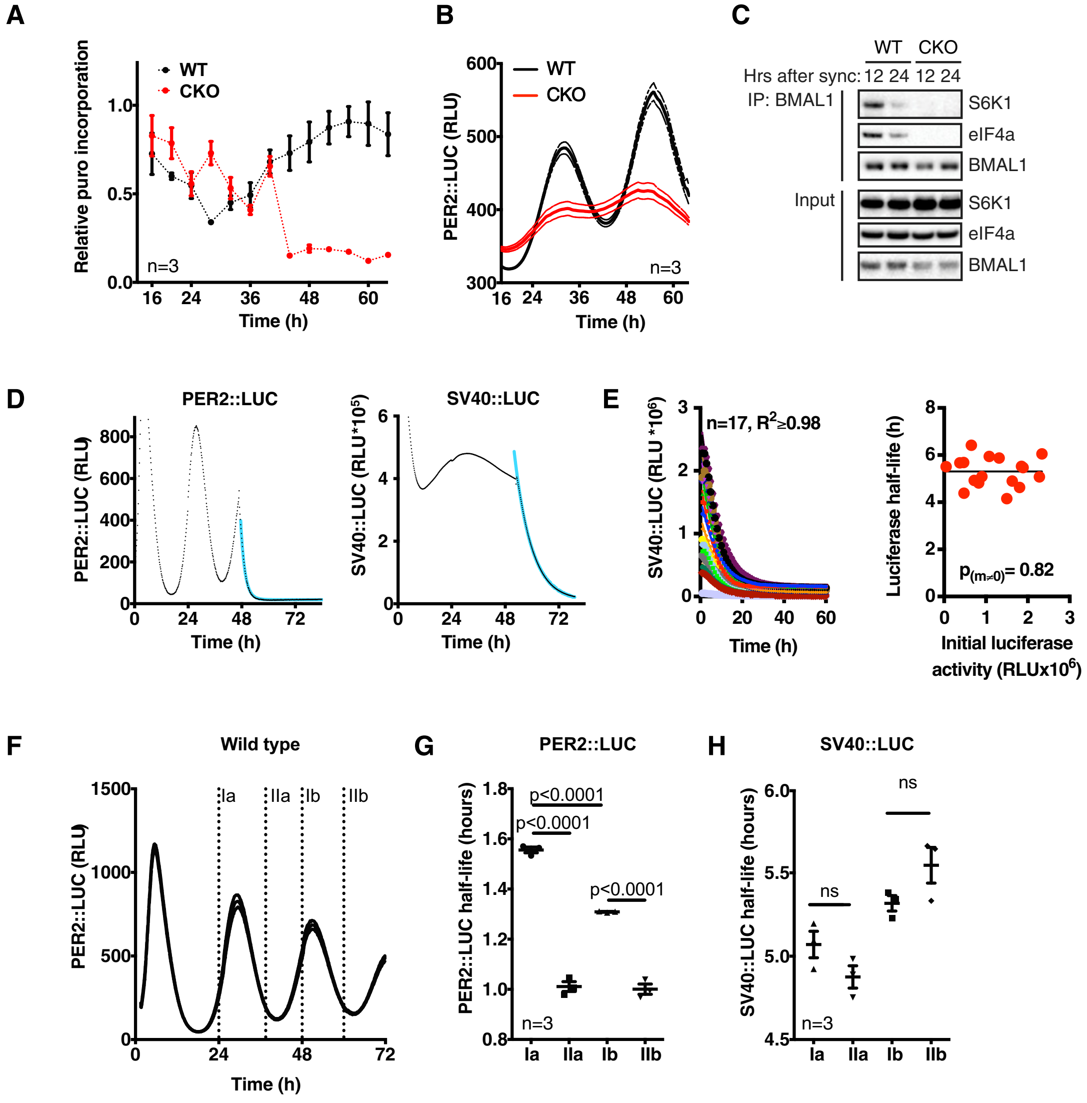

Figure EV4. PER2::LUC stability oscillates in CRY-deficient cells.

A WT and CKO cells were assayed for puromycin incorporation over two circadian cycles. Cells were synchronised by temperature cycles and dexamethasone, and harvested every 3 h after a 10-min puromycin pulse (10 μg/ml). Incorporation was measured by Western blotting with an anti-puromycin antibody. Western blots were quantified and corrected for total protein loading (Coomassie staining). Mean ± SEM (n = 3).

B Bioluminescence co-recording of puromycin labelling time course shows circadian PER2::LUC expression in both genotypes. Mean ± SEM (n = 3).

C Western blot analysis of BMAL1 immunoprecipitation with antibodies specific for S6K, eIF4a and BMAL1. Cells were harvested 12 or 24 h after dexamethasone synchronisation, and BMAL1 was immunoprecipitated.

D Example of a bioluminescence recording of WT PER2::LUC (left) or SV40::LUC (right) cells pulsed with 10 μM CHX after 46 h of recording. The resulting raw data (symbols) were fitted with a one-phase decay curve (blue line).

E Multiple stable SV40::LUC fibroblast lines with different basal expression levels were treated with 25 μg/ml CHX allowing the turnover of luciferase to be inferred from the decay in bioluminescence signal. Left panel, the decline in luciferase activity was fit with a simple one-phase exponential decay curve (solid lines) to derive the half-life of luciferase in each cell line. Right panel, no significant relationship between the level of luciferase expression and luciferase stability was observed (straight line vs. horizontal line fit P-value is reported, extra sum-of-squares F-test).

F Timing of CHX pulses (labelled I-II a (cycle 1) and b (cycle 2)), plotted on bioluminescence trace of WT PER2::LUC control cells, corresponding to data presented in G and H.

G PER2::LUC half-life at different phases in the circadian cycle in WT cells (mean ± SEM). P-values were calculated by two-tailed t-test. Half-life was quantified by one-phase decay line-fitting of bioluminescence traces from CHX pulsed cells.

H SV40::LUC half-life at different phases in the circadian cycle in fibroblasts (mean ± SEM). P-values were calculated by two-tailed t-test.

Source data are available online for this figure.

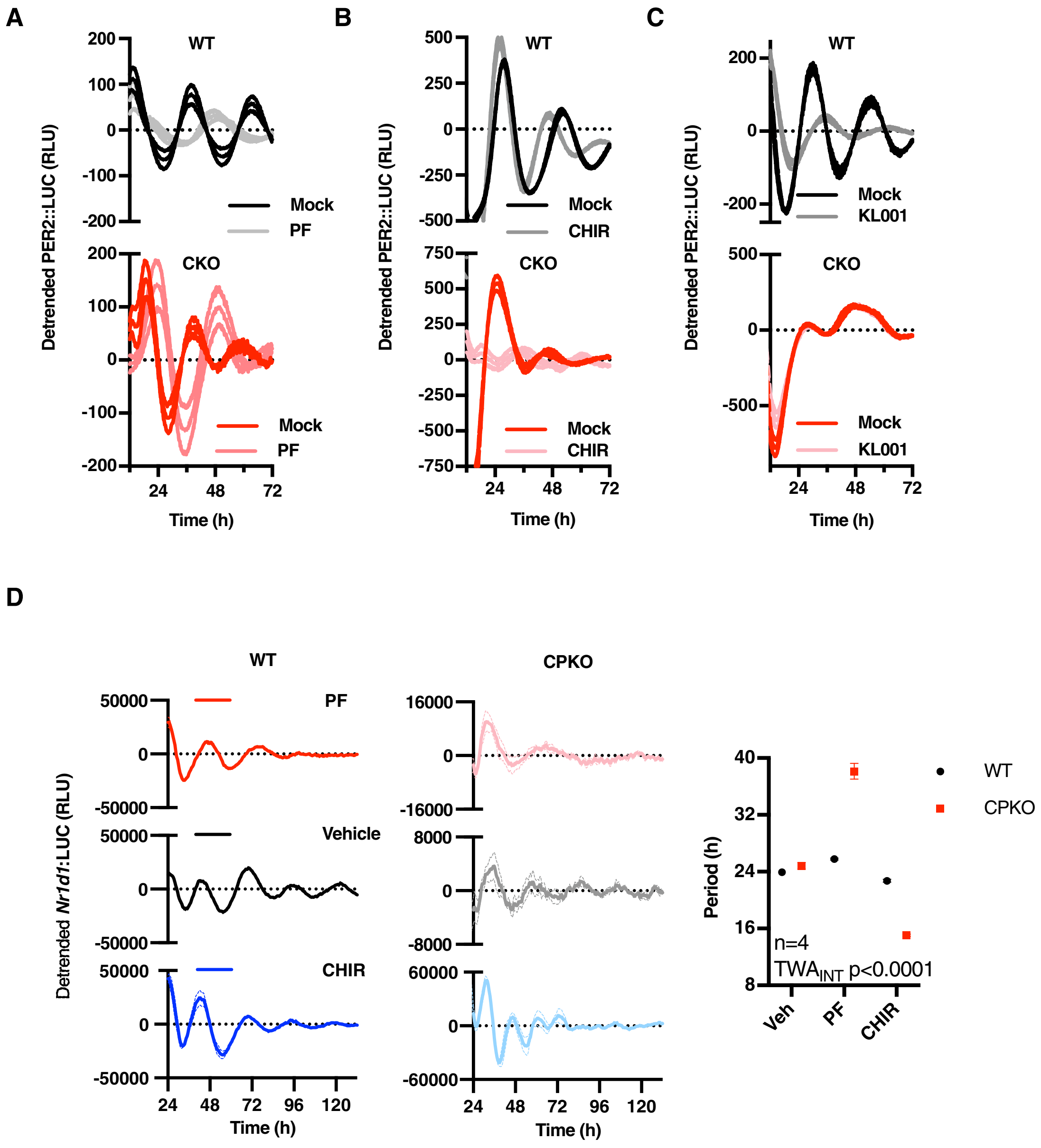

Figure EV5. A role for CK1 and GSK3 in the cytoplasmic oscillator.

A Bioluminescence recordings of WT and CKO PER2::LUC cells in the presence or absence of CK1δ/ε inhibitor PF670462 (0.3 μM; PF), as quantified in Fig 5A ($n = 3$, detrended mean ± SEM).

B As in (A), GSK3 inhibitor CHIR99021 (5 μM; CHIR).

C As in (A), in the presence of CRY turnover inhibitor KL001 (1 μM).

D Bioluminescence recordings and respective period quantifications of WT and CPKO *Nr1d1*::LUC cells in the presence or absence of CK1δ/ε inhibitor PF670462 (0.1 μM) or GSK3 inhibitor CHIR99021 (3 μM; CHIR) ($n = 4$, detrended mean ± SEM). *P*-value of the two-way ANOVA (genotype vs. drug interaction effect) is reported.

© 2021 The Authors
