# Supplementary figures and images for: CRYPTOCHROMES confer robustness, not rhythmicity, to circadian timekeeping
Source: EMBO J. 2021 Jan 25;40(7):e106745. doi: 10.15252/embj.2020106745 (PMC8013833; doi:10.15252/embj.2020106745)

SOURCE DATA EV1

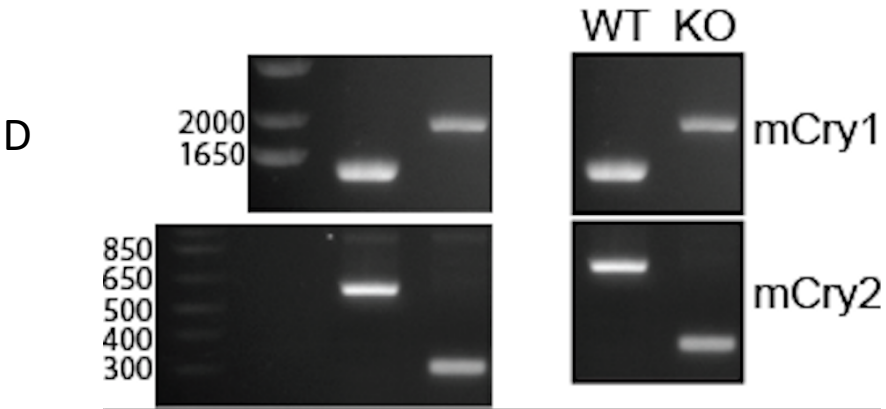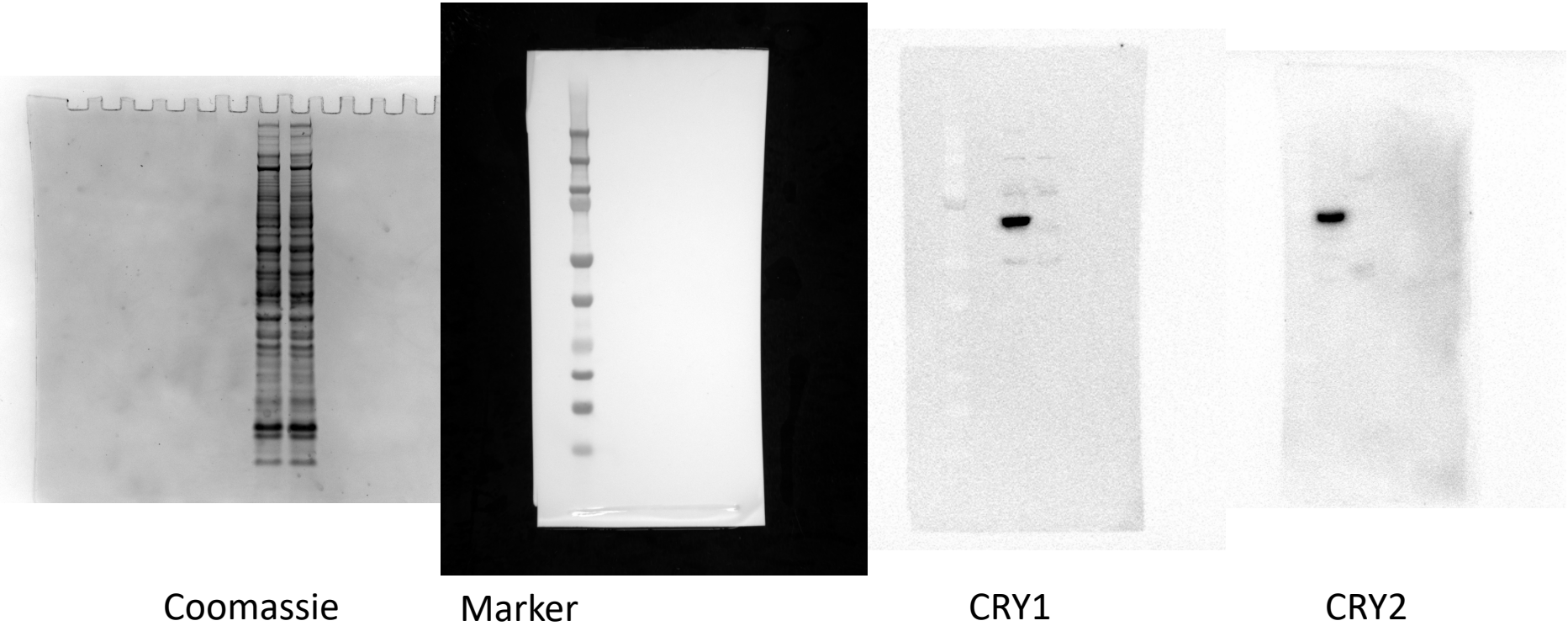

Supplement: Supplementary file 3 — Source Data for Expanded View [file EMBJ-40-e106745-s003.zip › embj2020106745-sup-0003-SDataFigEV1.pdf]

SOURCE DATA FIGURE EV3

B

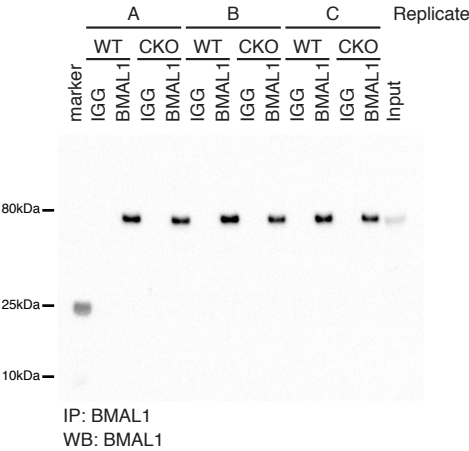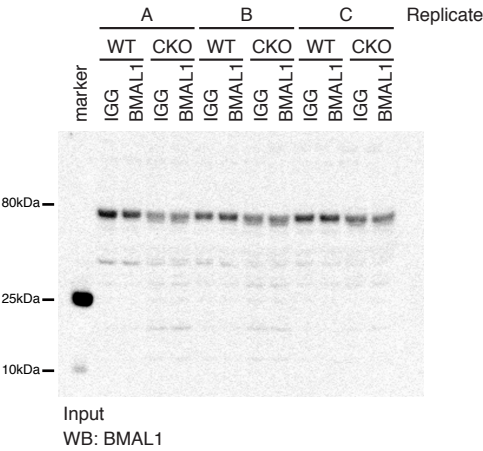

Supplement: Supplementary file 3 — Source Data for Expanded View [file EMBJ-40-e106745-s003.zip › embj2020106745-sup-0004-SDataFigEV3.pdf]

A

Replicate 1

Replicate 2

Replicate 3

WT

CKO

WT

CKO

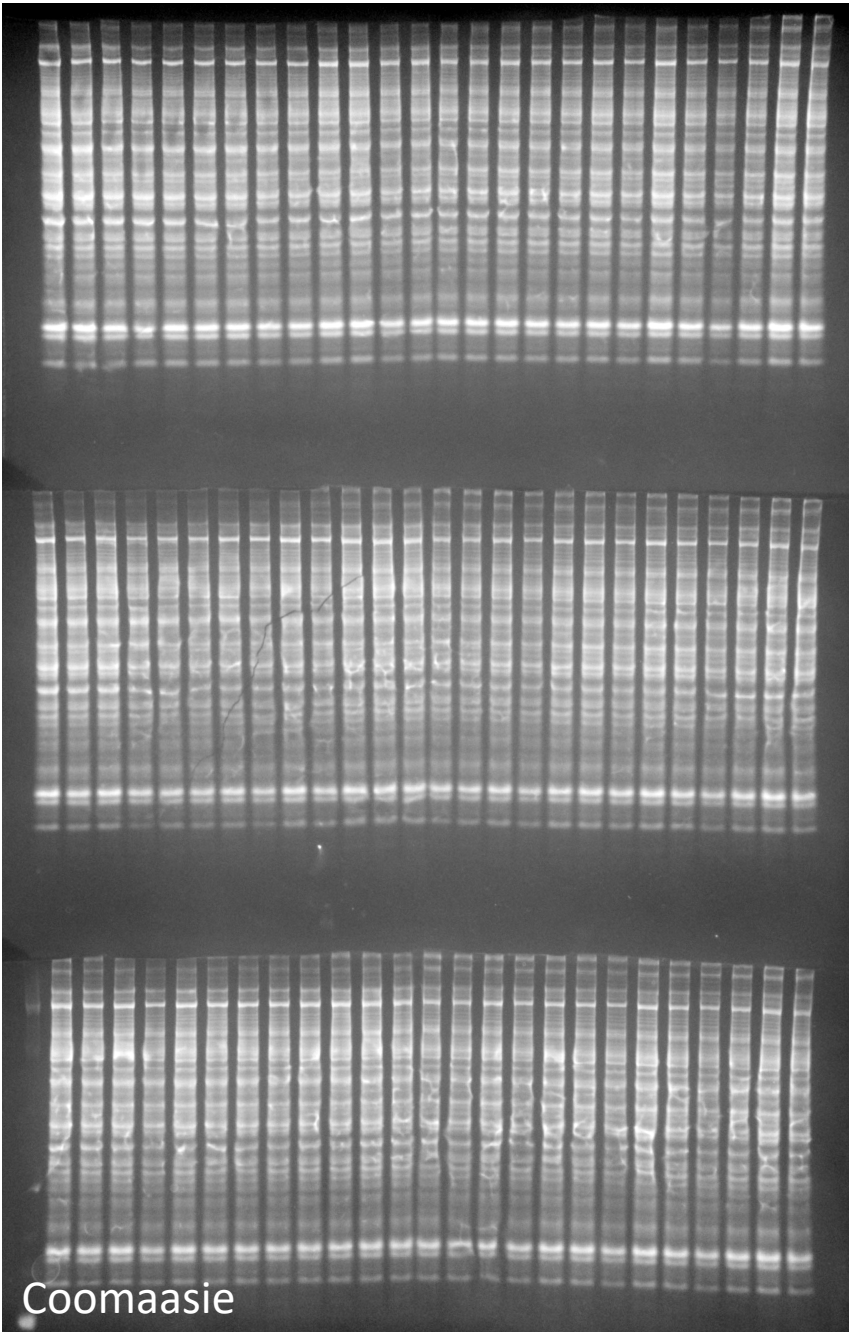

Coomaasie

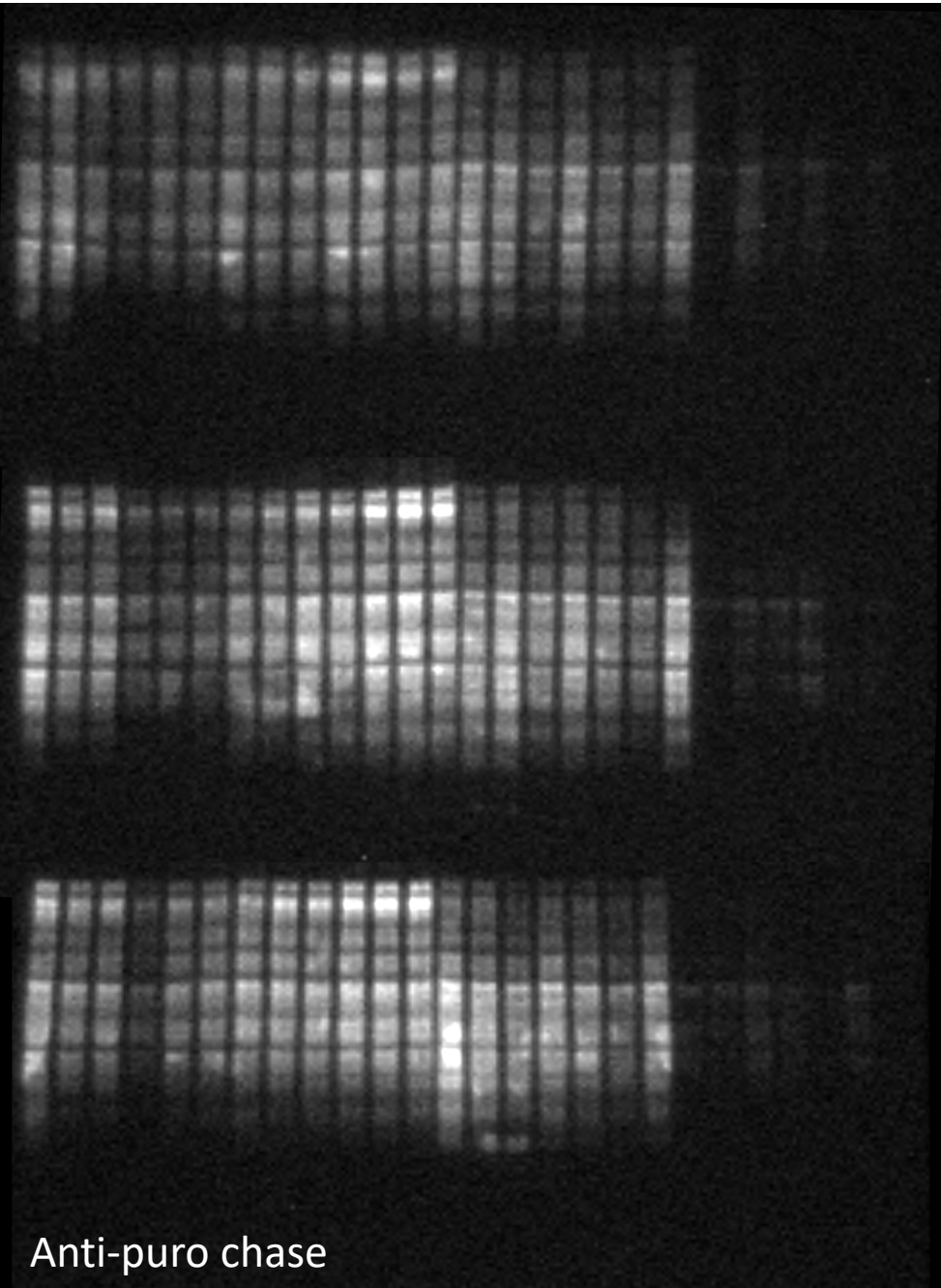

Anti-puro chase

Supplement: Supplementary file 3 — Source Data for Expanded View [file EMBJ-40-e106745-s003.zip › embj2020106745-sup-0005-SDataFigEV4.pdf]
